# Serological Evidence of Exposure to Saint Louis Encephalitis and West Nile Viruses in Horses of Rio de Janeiro, Brazil

**DOI:** 10.3390/v14112459

**Published:** 2022-11-06

**Authors:** Flávia Löwen Levy Chalhoub, Marco Aurélio Pereira Horta, Luiz Carlos Junior Alcantara, Alejandra Morales, Lilha Maria Barbosa dos Santos, Vinícius Guerra-Campos, Cintia D. S. Rodrigues, Carolina C. Santos, Maria Angélica M. Mares-Guia, Alex Pauvolid-Corrêa, Ana Maria Bispo de Filippis

**Affiliations:** 1 Laboratório de Flavivírus, Oswaldo Cruz Foundation, Rio de Janeiro 21040-900, Brazil; 2 Biosafety Level 3 Facility (BSL-3), Fundação Oswaldo Cruz, Rio de Janeiro 21040-900, Brazil; 3 Instituto Nacional de Enfermedades Virales Humanas, Pergamino 2700, Argentina; 4 Laboratório de Virologia Animal, Setor de Medicina Veterinária Preventiva e de Saúde Pública do Departamento de Veterinária da Universidade Federal de Viçosa (UFV), Viçosa 36570-900, Brazil

**Keywords:** West Nile virus, Saint Louis encephalitis virus, horses, plaque reduction neutralization test, RT-qPCR, Rio de Janeiro, Brazil

## Abstract

Infections with arboviruses are reported worldwide. Saint Louis encephalitis (SLEV) and West Nile viruses (WNV) are closely related flaviviruses affecting humans and animals. SLEV has been sporadically detected in humans, and corresponding antibodies have been frequently detected in horses throughout Brazil. WNV was first reported in western Brazil over a decade ago, has been associated with neurological disorders in humans and equines and its prevalence is increasing nationwide. Herein, we investigated by molecular and serological methods the presence or evidence of SLEV and WNV in equines from Rio de Janeiro. A total of 435 serum samples were collected from healthy horses and tested for specific neutralizing antibodies by plaque reduction neutralization test (PRNT_90_). Additionally, serum and central nervous system samples from 72 horses, including horses with neurological disorders resulting in a fatal outcome or horses which had contact with them, were tested by real-time reverse transcription–polymerase chain reaction (RT-qPCR) for both viruses. Adopting the criterion of four-fold antibody titer difference, 89 (20.4%) horses presented neutralizing antibodies for SLEV and five (1.1%) for WNV. No evidence of SLEV and WNV infection was detected by RT-qPCR and, thus, such infection could not be confirmed in the additional samples. Our findings indicate that horses from Rio de Janeiro were exposed to both SLEV and WNV, contributing to the current knowledge on the distribution of these viruses flaviviruses in Brazil.

## 1. Introduction

Emerging and reemerging viruses are mostly zoonotic, and among these, viruses transmitted by hematophagous arthropods (arboviruses) are of major importance, affecting both animals and humans [1]. Arboviruses are maintained in nature through sylvatic and urban transmission cycles involving vertebrates, as amplifying hosts, and hematophagous arthropods, as vectors [2].

The *Flaviviridae* family comprises the genera *Flavivirus*, *Pestivirus*, *Hepacivirus* and *Pegivirus*, but only *Flavivirus* contains arboviruses. West Nile virus (WNV) and Saint Louis encephalitis virus (SLEV) are species of the Japanese Encephalitis (JE) serocomplex [3] and are among the dozen flaviviruses of medical importance in Brazil [4,5].

SLEV and WNV are maintained in enzootic cycles of transmission mainly involving mosquito species of the genus *Culex*, as vectors, and some species of birds, as amplifying hosts [6,7]. Humans and domestic animals including equines are considered accidental or terminal hosts, not playing a common role in the maintenance cycle in nature. As such, they present a short and low-load viremia when infected, usually insufficient to act as source of infection for the main vectors [8,9]. Despite that, humans and equines are susceptible to clinical infection, which results in neurological disorder with a fatal outcome in some cases [10,11,12,13]. SLEV has been associated with neurological disorders, mostly in the United States and Argentina [14,15]. WNV is involved in human and equine neurological outbreaks throughout the world, with most cases being reported in the United States [16].

SLEV is an arbovirus native to the Americas and was first reported in the 1930s during a human outbreak of neurological disorder in Saint Louis, Missouri, United States [17,18]. Currently, SLEV is widespread in the Americas, from Canada to southern Argentina [19,20]. In Brazil, SLEV was first detected in mosquitoes collected in the 1960s on the Belém–Brasília highway [21]. Since then, SLEV has been isolated in different regions of the country from rodents, birds and sentinel rats [22]. SLEV has been associated with mild clinical cases of infection in humans from Pará, in north Brazil, [23] to São Paulo, in southeast Brazil [24]. In the 1970s, antibodies against SLEV were detected in children from rural areas of the state of Rio de Janeiro (RJ) [25]. SLEV has not been commonly reported to cause infection in vertebrates other than humans; however, in 2013 in southeast Brazil, it was isolated from the brain of a horse with neurological signs [26]. Several serological surveys conducted since 2005 in equines from Brazil suggest that horses have been frequently exposed to SLEV throughout the country [27,28,29,30,31,32,33].

The first evidence of WNV circulation in Brazil emerged in 2011 when specific neutralizing antibodies were detected in healthy horses from the Pantanal, a South American floodplain located in western Brazil [34]. In 2014, reports on the circulation of WNV in Brazil intensified, with serological and clinical evidence of WNV infection reported in a farmer with a neurological disorder in the state of Piauí, in northeast Brazil [35]. In 2018, WNV was detected in tissue samples of horses with neurological disease in the state of Espírito Santo in southeast Brazil [36,37]. Recently, similar reports emerged from two other states, including Minas Gerais and São Paulo from the southeast region of the country [38,39]. Furthermore, WNV was detected in the states of Ceará and Rio Grande do Sul, located in the northeast and south regions of the country, respectively [33,40]. Over a decade after the first report on its circulation in Brazil, WNV has been evidenced in all regions of the country [30,33,41,42,43].

Brazil has environmental and socioeconomic characteristics ideal for the circulation and spread of arboviruses [44,45,46]. Peculiar characteristics that favor a diverse set of mosquito populations, including species that are vectors for human and animal pathogens are seen in RJ [47]. The metropolitan area of RJ has long been affected by outbreaks of *Aedes*-borne arboviruses [48,49,50], and was recently hit by sylvatic yellow fever that is maintained by sylvatic *Haemagogus* spp. and *Sabethes* spp. mosquitoes [51]. RJ is divided into six mesoregions, each possessing different climatic, economic and social characteristics [52]. In North Fluminense and Coast mesoregions, four important areas with concentrations of migratory birds exist [53], which can be of particular interest for investigation of arboviruses maintained in bird–mosquito cycles of transmission [54].

The exposure of horses from RJ to flaviviruses as SLEV and WNV has been rarely assessed and results of mostly based on ELISA serosurveys are ambiguous. In a ELISA-based serosurvey conducted in the South Fluminense mesoregion, no evidence of WNV was found in local horses [30]. In our preliminary investigation, using a similar approach we found serological evidence of WNV and SLEV in horses using a blocking-ELISA [55]. Thus, the main purpose of the present study was to confirm by highly specific serological and molecular methods the circulation and exposure of horses in RJ, southeast Brazil to WNV and SLEV.

## 2. Materials and Methods

The study was approved by the Animal Ethics Committee of Fundação Oswaldo Cruz License (CEUA-Fiocruz, 07.2016 protocol 047.2015) in compliance with the requirements of Brazilian Law 11794.2008, which regulates the scientific use of animals, including the principles of the Brazilian society of science in laboratory animals.

### 2.1. Sampling

For the present study, blood samples were collected from horses of stud farms, ranches and horse training centers located in different mesoregions of RJ. Additionally, we also collected and tested samples from horses that presented acute neurological syndrome without a defined cause, and horses that had close contact with sick.

#### 2.1.1. Seroprevalence Study

##### Sample Size

The sample size for the serological evaluation was calculated based on the expectance of detection of at least one WNV- or SLEV-seropositive horse through the software OpenEpi [56]. We used the number of horses (heads) reported by the Brazilian Institute of Geography and Statistic Automatic Recovery System—SIDRA, 2017 [57] as a reference for the horse population in RJ, estimating the frequency of seropositivity at 50% (±5%) and a confidence limit of 5%. To obtain a confidence interval of at least 95%, 383 animals should be evaluated. In the present study, to cover all climatic, economic and social conditions in RJ, samples from 435 horses from different regions were evaluated. Sample collections were divided between six geographic mesoregions. The proportion of horses from each mesoregion reported to the population of the state was used to calculate the percentage of samples that should be collected from each mesoregion.

##### Study Area

**Figure 1:**
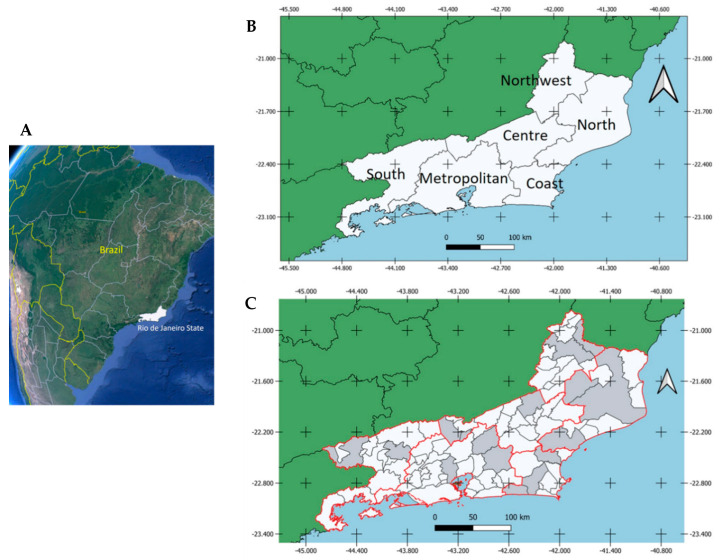
(**A**) Geographic location of RJ (white) in Brazil; (**B**) Geographic mesoregions of RJ; (**C**) Municipalities within RJ where horses were sampled (gray) between August 2015 and March 2017 and tested for WNV and SLEV.

Blood samples from 435 healthy horses were collected between August 2015 and March 2017 from 21 equine properties located in 16 municipalities of six different mesoregions of RJ. At least two municipalities within each mesoregion were selected. These mesoregions included Metropolitan, Northwest Fluminense, North Fluminense, Coast, Centre Fluminense and South Fluminense (Table 1, Figure 1). During the sampling, variables such as health status, breed, function, sex, age and travel records were collected from owners or workers in charge through a comprehensive questionnaire. Only horses with no history of travel outside their mesoregion were included in the study.

The 435 blood samples were collected from the jugular vein of healthy animals in tubes without anticoagulants and transported to the Laboratório de Flavivírus at Fundação Oswaldo Cruz (Fiocruz), the regional center of reference for arbovirus diagnostics belonging to the national network of laboratories of the Brazilian Ministry of Health. Blood samples were then centrifuged, and serum collected and kept at −70 °C.

Mesoregions sampled included Metropolitan (114 samples), Northwest Fluminense (80 samples), North Fluminense (76 samples), Coast (76 samples), Centre Fluminense (49 samples) and South Fluminense (40 samples) (Table 1).

##### Horse Categories

Horses were classified in categories according to their activities, including sport, recreation, reproduction and work. Animals were also divided by age groups including Group 1 (≤6 months old), Group 2 (>6 months and ≤24 months old), Group 3 (>24 months and ≤72 months old), Group 4 (>72 months and ≤120 months old) and Group 5 (>120 months old).

#### 2.1.2. Horses with Neurological Disorder

A total of 72 samples from 30 horses that presented acute neurological syndrome without a defined cause, including 11 dead horses and 42 samples from equines that had close contact with sick equines, were collected from seven properties in RJ between August 2015 and May 2021. All animals had a history of vaccination for at least one of the following viruses: rabies, Madariaga, Western equine encephalitis, equid influenza and herpesvirus.

Sampled municipalities included Teresópolis, Saquarema, Friburgo, Duas Barras, Maricá and Rio de Janeiro (Table 2). Some properties located in the municipalities of Saquarema, Duas Barras and Friburgo reported cases of rabies among the horses with neurological disorders. Clinical manifestations included photosensitivity, incoordination, disorientation, ataxia in limbs, muscle tremors, exophthalmos, loss of visual ability and pedaling movements.

Different types of clinical samples were collected (Table 2). Blood samples were collected from the jugular vein and cerebrospinal fluid (CSF) from lumbosacral space, both in tubes without anticoagulants. Eleven cases were fatal, and from these, tissues from the central nervous system (CNS) including the brain and spinal cord were collected. Samples were refrigerated and transported to the laboratory, where they were kept at −70 °C.

### 2.2. Laboratory Tests

#### 2.2.1. Plaque Reduction Neutralization Test (PRNT_90_)

Serum samples of healthy horses were tested for WNV and SLEV using the plaque reduction neutralization test (PRNT). Shortly thereafter, the presence of specific neutralizing antibodies in serum samples was determined by screening followed by endpoint titer PRNT, as previously described [58].

Samples were heat-inactivated in a water bath at 56 °C for 30 min. After that, samples were two-fold serially diluted in medium 199 containing 5% bovine fetal serum. Viral stock of SLEV and WNV were diluted. Diluted samples were mixed in equal volume of viral suspension containing ~80 plaque forming units (PFU), and then incubated for 1 h at 37 °C. After incubation, 100 µL serum and virus solution was inoculated in six-well plates with VERO cells (ATCC CCL-81) and further incubated for 1 h at 37 °C in 5% CO_2_ atmosphere. Cell plates inoculated with viral suspensions were assigned as viral control and used to determine the percentage of virus neutralization from each sample. Cell plates inoculated with viral suspension and diluted sera were overlaid by a 0.5% agarose solution. Overlaid plates were incubated for four days and then overlaid again with a neutral red agarose solution. The next day, the number of plaques was counted and compared to the viral control. Serum samples were initially screened at a single dilution (1:10). Samples that neutralized 90% or more plaques (PRNT_90_) were then tested in duplicate at higher dilutions, in serial two-fold dilutions from 1:10 to 1:2560. The endpoint titers were determined as the reciprocal of the highest dilution that had 90% or more plaques neutralized. Archived serum samples of horses that were seropositive and seronegative for SLEV and WNV in previous studies were used as positive and negative controls, respectively.

Serum samples that presented PRNT_90_ titers equal to or higher than 10 for WNV and SLEV were then sequentially tested for the following other flaviviruses: Zika (ZIKV), Dengue (DENV-1) and Ilheus (ILHV), as previously described [28,32].

**Figure 2:**
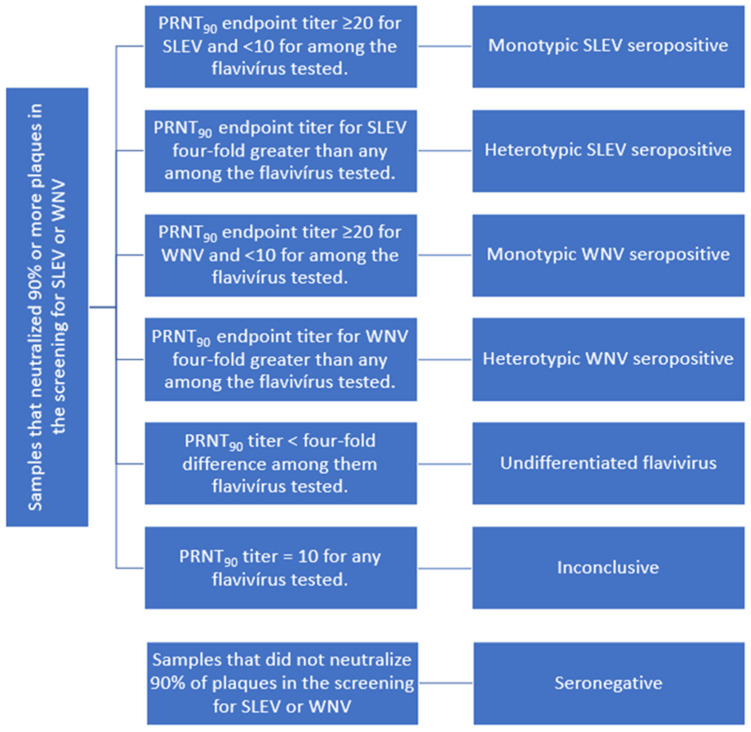
Seropositivity criteria used for SLEV and WNV in horses from RJ.

Samples with PRNT_90_ titer equal to or higher than 20 for WNV or SLEV and <10 for all other flaviviruses tested were considered seropositive in monotypic reactions. Additionally, samples that presented heterologous reactions, with PRNT_90_ titers for SLEV or WNV at least four times higher than that observed for the other flaviviruses tested, were also considered seropositive [32]. Samples that presented a titer less than four-fold greater for any flavivirus were considered seropositive for undifferentiated flaviviruses. Samples that presented PRNT_90_ titer 10 for any flavivirus tested—SLEV, WNV, DENV-1, ILHV or ZIKV—were considered inconclusive. Samples with PRNT_90_ < 10 for all flaviviruses were considered seronegative (Figure 2).

#### 2.2.2. Horses with Neurological Disorder

Sera, cerebrospinal fluid and CNS tissues from equines with neurological disorder were tested by real-time reverse transcription–polymerase chain reaction (RT-qPCR) for WNV and SLEV. Equines that had close contact with sick equines were also sampled and tested. In brief, samples of solid tissues (30 mg) were macerated in 600 μL of lysis buffer, centrifuged and the supernatant submitted to RNA extraction using QIAamp Viral RNA Mini Kit (Qiagen, Hilden, Germany) according to the manufacturer’s instructions. Extracted RNA was then tested by specific RT-qPCR for WNV and SLEV based on the amplification of a region of the envelope gene, as previously described [59,60].

### 2.3. Statistical Analysis

Categorical variables including seropositivity for WNV and SLEV in horses, mesoregion, function, sex and horses’ age groups are displayed as numbers and percentages (%). We chose to examine the relation between seropositivity and the other categorical variables with Pearson’s Chi-squared test and Fisher’s exact row test to compare the prevalence between groups (screenings and final PRNT results). Statistical significance was defined as values of two-tailed *p*  <  0.05. All analyses were performed using RStudio Software (version 2021.09.2) [61] and Excel version 2016 (Microsoft, Redmond, WA, USA). Frequencies and seroprevalences were mapped using QGIS (version 3.22).

## 3. Results

A total of 435 healthy horses were sampled between August 2015 and March 2017 in 16 municipalities of six different mesoregions of RJ (Figure 2). Healthy horse samples, then tested by specific serological and molecular methods for WNV and SLEV.

No animal had a history of journey outside of the given mesoregion where they were sampled. Furthermore, despite their existence and use since 2002 in some parts of the world, no vaccines for WNV are licensed in Brazil by the Office Veterinary Service. Thus, no animal had history of vaccination for WNV.

### 3.1. Sampling and Zootechnical Features

Of the 435 healthy horses sampled, 71.3% (*n* = 310) were female and 28.7% (*n* = 125) male. The horses were classified according to their activities: 218 (50.1%) individuals were for sport, 40 (9.2%) for recreation, 175 (40.2%) for reproduction and 2 (0.5%) for work.

Furthermore, healthy horses were divided into five groups by age: 7 (1.6%) belonged to Group 1 (up to 6 months old), 63 (14.5%) to Group 2 (between 7 and 24 months old), 130 (29.9%) to Group 3 (between 25 and 72 months old), 141 (32.4%) to Group 4 (between 73 and 120 months old), and 94 (21.6%) to Group 5 (animals over 120 months old).

A total of 72 serum samples were collected from the horses with history of neurological disorder or those in contact with such horses. Other samples from these horses included six samples of CNS tissues, five of CSF and six of spinal cords (Table 2). Among these horses, 23 (31.9%) individuals were females and 49 (68%) males. Regarding activity, 61 (84.7%) were classified as “for sport” and 11 (15.3%) “for reproduction”. These samples were tested only by RT-qPCR.

### 3.2. PRNT_90_ Results

Among the 435 screened healthy horses, 38% (*n* = 165) presented PRNT_90_ ≥ 10 for WNV and/or SLEV and the corresponding samples were selected for determining endpoint titers, while 62% (*n* = 270) presented PRNT_90_ < 10 and were considered seronegative.

Among the samples that were reactive in the screening, 32.9% (*n* = 143) were reactive for SLEV, with 19.3% (*n* = 84) being monotypic for SLEV. Regarding WNV, 18.4% (*n* = 80) were reactive in the screening and among these, 4.8% (*n* = 21) had WNV monotypic results. A total of 13.5% (*n* = 59) of all serum samples tested presented seropositivity for both SLEV and WNV.

Among individuals reactive in the screening, 21.6% (*n* = 94) were seropositive for SLEV (20.4%, *n* = 89) or WNV (1.1%, *n* = 5). A total of 7.4% (*n* = 32) of samples were seropositive for an undifferentiated flavivirus, and 6.4% (*n* = 28) of samples were inconclusive. Among the samples that were seropositive for SLEV or WNV, 70.8% (*n* = 63) and 60.0% (*n* = 3), respectively, were monotypic results. From the total of 435 samples, 14.5% (*n* = 63) were seropositive for SLEV and 0.7% (*n* = 3) were seropositive for WNV.

**Figure 3:**
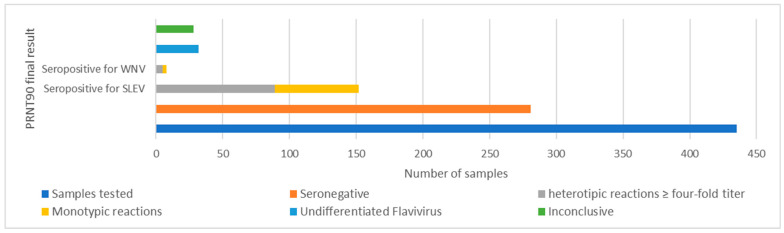
PRNT_90_ results for SLEV and WNV in horses evaluated from RJ.

From the total of 435 tested samples, 64.6% (*n* = 281) were seronegative. These samples were also seronegative for ZIKV, DENV-1 and ILHV (Figure 3, Table 3).

A total of 89 serum samples that were seropositive for SLEV had serum neutralizing antibody titers ranging from 20 to 320, with 70.7% (*n* = 63) monotypic and 34.9% (*n* = 26) heterotypic reactions. Of the 63 samples with monotypic reaction for SLEV, 22 had a neutralizing antibody titer of 20, 16 a titer of 40, 13 a titer of 80, 10 a titer of 160 and two samples presented titer of 320. Among the 26 heterotypic samples, the titers ranged between 40 (*n* = 9), 80 (*n* = 11), 160 (*n* = 5) and 320 (*n* = 1). Furthermore, 23% (*n* = 6) of them presented anti-WNV antibody titers ≥ 20, one sample with 80, another with 40 and four samples with 20 titer. The others 20 samples had titers lower than 20 (Table S1).

The five samples seropositive for WNV had serum neutralizing antibody titers of 20 (*n* = 2), 320 (*n* = 2) and 2560 (*n* = 1), with 60% (*n* = 3) monotypic and 40% (*n* = 2) heterotypic reactions. Serum specimens from the two horses with serum antibody titers of 40, as well as one of the two samples that had a titer of 320, had monotypic reactions for WNV. The other serum samples with a titer of 40, one with a titer of 2560 and one with a titer of 10 for SLEV had heterotypic reactions.

A total of 32 samples were seropositive for undifferentiated flavivirus; of these, 18 (56.2%) had antibody titers ≥ 20 for SLEV, and 15 (46.8%) for WNV. Serum neutralizing antibody titers were: 10 (*n* = 14), 20 (*n* = 7) and 40 (*n* = 11) for SLEV; and 10 (*n* = 17), 20 (*n* = 10), 40 (*n* = 2) and 80 (*n* = 3) for WNV (Table S2).

#### Results by Geographic Location, Sex, Activities and Age Group

**Figure 4:**
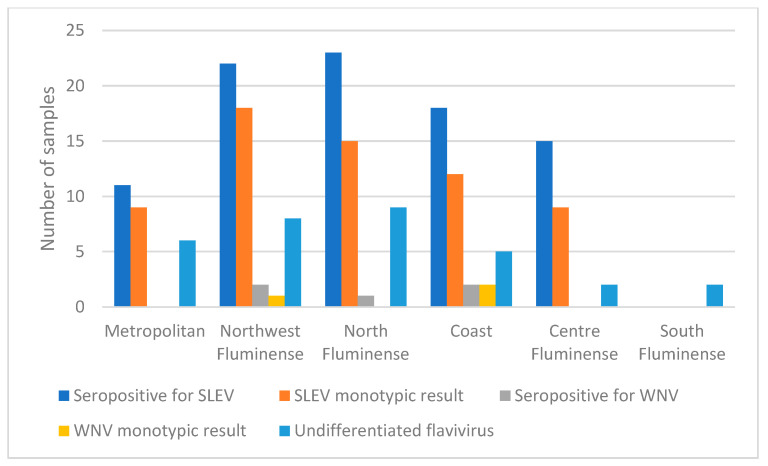
Comparative results of samples seropositive for WNV and SLEV in mesoregions of RJ, by PRNT_90_.

Mesoregions that had SLEV-seropositive equines were North Fluminense with 30% (*n* = 23), Northwest Fluminense with 27.5% (*n* = 22), Coast with 23.7% (*n* = 18), Centre Fluminense with 30.6% (*n* = 15) and Metropolitan with 9.6% (*n* = 11). The differences between regions were statistically significant (*p* < 0.001). In these regions, the corresponding seropositive samples with monotypic reactions were: 65.2% (*n* = 15), 81.8 % (*n* = 18), 66.6% (*n* = 12), 60% (*n* = 9) and 81.8% (*n* = 9), respectively. In all municipalities where samples were collected from, except for São Fidélis in the North Fluminense region, there were positive monotypic and heterotypic results for SLEV (Figure 4).

The five samples seropositive for WNV had monotypic antibodies and four-fold greater antibody titer for this virus than for others flaviviruses that were tested for, and were distributed in four municipalities of three mesoregions: Macaé in North Fluminense with 1.3 % (*n* = 1), Bom Jesus do Itabapoana in Northwest Fluminense with 2.5% (*n* = 2) and Saquarema and Araruama in Coast mesoregion with 2.5% (*n* = 2). The differences between regions were not statistically significant (*p* = 0.39). The monotypic reactions among the seropositive samples in each mesoregion were 50% (*n* = 1) in Northwest Fluminense, São Fidélis and 100% (*n* = 2) in the Coast mesoregion in Saquarema and Araruama (Figure 4, Table 3).

**Figure 5:**
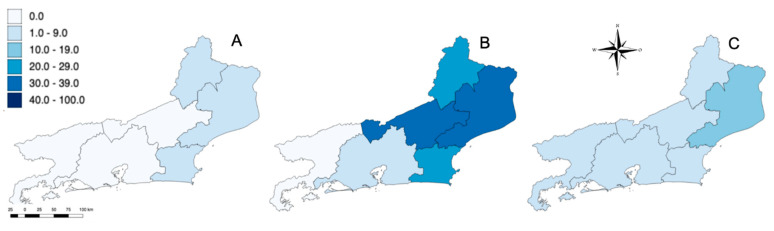
Location of equines that were seropositive (using PRNT_90_) for WNV (**A**), SLEV (**B**) and undifferentiated flavivirus (**C**) in RJ. Higher color intensity indicates higher seroprevalence (%).

Horses seropositive for undifferentiated flavivirus were found in all mesoregions sampled including North Fluminense with 11.8% (*n* = 9), Northwest Fluminense with 10.0% (*n* = 8), Coast with 6.6% (*n* = 5), Centre Fluminense with 4.1% (*n* = 2), Metropolitan with 5.3% (*n* = 6) and South Fluminense with 5.0% (*n* = 2); the differences were not statistically significant (*p* = 0.42) (Figure 4 and Figure 5, Table 3). The South Fluminense mesoregion was the only one that had no equines seropositive for SLEV and WNV.

Regarding horse function, of 89 SLEV-seropositive horses, 49% (*n* = 44) were classified “for reproduction”, 42% (*n* = 37) “for sport”, and 9% (*n* = 8) “for recreation”. Neither of the two horses classified as “for work” were seropositive for SLEV (Table 3). Of 175 horses classified as “for reproduction”, 25.1% (*n* = 44) were seropositive for SLEV, with 63.6% (*n* = 28) presenting monotypic reactions. Meanwhile, 20% (*n* = 8) and 17% (*n* = 37) of the horses classified as “for recreation” (*n* = 40) and “sport” (*n* = 218), respectively, were seropositive for SLEV. Seropositive samples had 87.5% (*n* = 7) and 75.6% (*n* = 28) monotypic reactions, respectively. The seroprevalence for SLEV was substantially higher in reproduction horses when compared to work horses although the difference was not statistically significant (*p* = 0.21). However, of 175 horses classified as “for reproduction”, only 2.3% (*n* = 4) were seropositive for WNV, with 50% (*n* = 2) having monotypic reactions. From the two horses classified as “for work”, one was seropositive (50%) and had a monotypic reaction (*n* = 100%).

The seroprevalence for SLEV was 2.2% (*n* = 2) in Group 1, 12.3% (*n* = 11) in Group 2, 22.4% (*n* = 20) in Group 3, 38.2% (*n* = 34) in Group 4 and 25% (*n* = 22) in Group 5 (*p* > 0.05). Within each group, horses with monotypic antibodies against SLEV were: 100% (*n* = 2) for Group 1, 90.9% (*n* = 10) for Group 2, 60% (*n* = 12) for Group 3, 70.5 % (*n* = 24) for Group 4 and 68.1% (*n* = 15) for Group 5. Among the horses seropositive for SLEV, 62 (70%) were female and 27 (30%) were male. Of all the 310 females tested, 62 (20%)—and of all the 125 male horses, 27 (21.6%)—were seropositive for SLEV. The difference between the two groups was not statistically significant (*p* > 0.05) (Table 3). Among the seropositive horses, 70.9% (*n* = 44) of the females and 70.3% (*n* = 19) of the males had monotypic reactions.

Five horses were seropositive for WNV; one (20%) was from the group of two horses classified as “for work” and four (80%) from the group of 175 horses classified as “for reproduction” (*p* > 0.05). Among these, two horses classified as “for reproduction” (50%) and one classified as “for work” (100%) had monotypic reactions. Regarding age, among the five WNV-seropositive horses, three (60%) were from Group 4 and two (40%) from Group 5 (*p* > 0.05), and within each group, 66.6% (*n* = 2) from Group 4 and 50% (*n* = 1) from Group 5 had a monotypic reaction for WNV. Of five horses that were seropositive for WNV, one (20%) was male and four (80%) were female. Among 125 males, 1 (0.8%) was seropositive for WNV, while among 310 females, 4 (1.3%) were seropositive for WNV (*p* > 0.05) (Table 3). Among the seropositive horses, two (50%) of the females and one (100%) of the males had monotypic reactions.

### 3.3. Real-Time Reverse Transcription–Polymerase Chain Reaction (RT-qPCR) Results

A total of 130 animals, including 32 horses with neurological disorder and 98 horses which had contact with them, tested negative for SLEV and WNV as assessed by RT-qPCR.

## 4. Discussion

The recent outbreaks of flaviviruses highlight their transmission potential and may be a cause for a dynamic state of emergence. Flaviviruses and other arboviruses need to be identified and continuously monitored as an instrumental public health strategy, which will provide us with measures to respond rapidly to emerging and re-emerging viral epidemics.

Human and equine cases of SLEV and WNV infection have been reported in Brazil [26,35,37,62,63]. Serosurveys of arboviruses conducted throughout the country have shown that healthy horses in different regions have been exposed to both flaviviruses [27,28,29,30,64,65]. Equines attract mosquito vectors and, therefore, are frequently exposed to mosquito-borne flaviviruses that trigger a humoral response, which ultimately makes equines an instrumental tool for enzootic arbovirus circulation [66]. In the present study, specific neutralizing antibodies for SLEV and WNV were assessed by PRNT_90_ in horses from different mesoregions of RJ. We used a conservative criterion of seropositivity to reduce the chances of cross-reactivity and the most specific serological method for the detection of flavivirus-neutralizing antibodies [67]. We report the titers using a 90% cutoff. In seropositive samples with a four-fold greater titer for one of the flaviviruses than for others, we additionally determined the monotypic responses, as previously reported [28].

We considered monotypic serologic responses to be the most reliable, as these samples reacted with just one of all viruses employed in the tests with no indication of cross-reaction. This stringent criterion is particularly valuable in areas where several flaviviruses co-circulate, because of the high potential for cross-reactivity mainly on secondary infections [68]. Samples that had a heterotypic response were classified as seropositive for SLEV or WNV, or as undifferentiated for flavivirus which could indicate more than one exposure in the animal.

The prevalence of undifferentiated flavivirus results can be attributed to the cross-reactive commonly observed among flaviviruses [69]. In addition, in individuals who are sequentially infected by a heterologous flavivirus species, the antibody levels against the original virus can increase, producing cross-reactive heterologous antibodies [70]. Considering the co-circulation of flaviviruses in RJ [48,49,50,51], the difference in antibody titers between the flaviviruses tested may have resulted in difference of less than four-fold, and thus, the respective sample may not have been classified as seropositive to a determined flavivirus. That would be the case especially among the samples that had high titers for both viruses, as we had in this study, but did not reach to the criteria threshold limit of four-fold titer.

Of the 32 horses seropositive for an undetermined species of flavivirus, 18 (56.2%) had antibody titers for SLEV ≥ 20, and 15 (46.8%) had antibody titers for WNV ≥ 20. Furthermore, these samples might have had contact with both viruses, SLEV and WNV. Among the 89 seropositive samples to SLEV, 26 (29.2%) had heterotypic reactions and their titers were 40 (*n* = 9), 80 (*n* = 11), 160 (*n* = 5) and 320 (*n* = 1). Most of these samples (*n* = 20) also had a low titer for WNV with only six having a titer ≥ 20, more precisely: 80 (*n* = 1), 40 (*n* = 1) and 20 (*n* = 4). The same happened with the heterotypic samples seropositive for WNV—one of them, with a high titer for WNV (2560), also had low titer for SLEV (10). Another one, with a titer of 320 for WNV, also presented a titer of 40 for SLEV, indicating an infection with SLEV. In all cases, some seropositive samples might have been overlooked in the seroprevalence calculation.

The seroprevalence for SLEV observed in the present study was higher than that observed for WNV. Eighty-nine (20.4%) equines were confirmed seropositive for SLEV, and five (1%) for WNV. Similar rates of infection with SLEV and WNV were reported by other studies applying the same conservative criterion of seropositivity [28]. Despite the differences in the diagnostic methods used in serosurveys conducted throughout the country, WNV has a lower prevalence than SLEV in various regions of Brazil [33]. A serological survey using in-house ELISA, assessing samples collected from horses in RJ between 2004 and 2009, found no seropositivity for IgG WNV and a prevalence of 6% for SLEV [30,68]. In a study using a similar diagnostic approach, the same seroprevalence for SLEV was found in horses from Minas Gerais, in the southeast of Brazil [31]. In Brazil, SLEV infection has been evidenced in humans for decades, and reports on its existence in equines have been increasing [25,26,27,28,29,62,63,71].

Regarding WNV, reports on its circulation throughout the country are emerging. If the increase of reports is related to a more sensitive surveillance or to a recent dissemination of WNV throughout the country remains unknown. Recent cases have been reported in humans and horses mainly from the northeast [32,35,40,41,72] and southeast [36,37,38,39] regions of the country, thus increasing its importance and visibility in Brazil. In one of the first serosurveys for WNV conducted in the Pantanal wetlands located in western Brazil, 3% of the studied equines presented neutralizing antibodies for WNV while over 5% were seropositive for SLEV [34]. In another study conducted in the same region, 8% of the equines presented neutralizing antibodies for WNV [42]. Between 2004 to 2009, 1% of the serum samples collected from horses from different states in Brazil were seropositive for WNV [68].

In 2018, WNV was detected in horses with neurological disorders in Espírito Santo, a state that geographically borders RJ [37]. The seroprevalence for WNV in RJ reported in the present study confirms the spread of WNV within the Brazilian territory. Considering the potential circulation of other flaviviruses in Brazil, which could ultimately result in false-positive results owing to cross-reactions, we included as differential diagnoses other flaviviruses commonly found in Brazil: ZIKV, ILHV and DENV-1 [32]. Although there is no information regarding equines acting as amplifying hosts of these arboviruses, they are exposed and mount humoral responses to these viruses [73,74]. In the present study, none of the equine samples presented neutralizing antibodies for ZIKV, ILHV or DENV-1. These results were expected for DENV and ZIKV despite their circulation during the period of sampling, as both arboviruses are transmitted by *Aedes aegypti*, a vector that feeds preferentially on humans. Conversely, ILHV, which is an enzootic flavivirus, has presented high seroprevalence in other serosurveys of arboviruses conducted on horses in western Brazil [28,65]. The negative results suggest no exposure of the tested horses to these flaviviruses and reduce the chances for false positive results owing to the detection of cross-reactive antibodies [73].

In this study, 435 samples were collected from horses in all the mesoregions of RJ, regions with different climatic, economic and social characteristics [52]. No horse included in this study had a history of traveling outside its mesoregion. Thus, viral prevalence could be correctly assigned to each mesoregion. The animals that presented neurological manifestations were not selected under the same criteria and were not included in the assessment of regional seroprevalence.

Despite variation in seropositivity for both viruses among the mesoregions, differences were statistically significant only for SLEV. The mesoregions with the highest seroprevalence for SLEV and WNV were Northwest Fluminense and Coast (for SLEV, *p* < 0.001). In the same mesoregions, including the north of RJ, we observed higher prevalence of monotypic reactions. Besides high humidity and temperature, both of these mesoregions are also characterized by recent increases in development and urbanization [75]. South Fluminense was the only mesoregion that had no evidence of SLEV or WNV infection and presented a low prevalence of undifferentiated flaviviruses. The WNV results in the South Fluminense mesoregion corroborate the findings presented by a previous ELISA-based study conducted with horses from the same mesoregion [30].

Regarding sex as a risk factor, we found no association between sex and seropositivity for SLEV (*p* > 0.05). The analysis of sex as risk factor for WNV infection was not performed because of the low number of samples.

The prevalence of SLEV infection was 25.1%, 20% and 17% in animals destined for reproduction, recreation and sports, respectively (*p* = 0.21). The difference in exposure to WNV between animals destined for work (50%) and reproduction (2.3%) was statistically significant (*p* < 0.001). The difference in seroprevalence between the two groups could be attributed to the management of horses belonging to each group. This variation could ultimately increase or decrease exposure to vectors, which is an important risk factor for arthropod-borne virus infections [44]. Furthermore, the distribution of samples collected between the categories was uneven, and some groups had a very small number of individuals when compared to others. For instance, only two horses were categorized as “for work”, while 218 individuals were classified as “for sport”. That factor may have impacted the statistical analysis of the differences in seroprevalence among the different categories of usage.

The seroprevalence for SLEV, WNV and undifferentiated flavivirus was directly proportional to the age group. Seroprevalence increased with age, which can be related to the longer exposure to both arboviruses during the lifetime. Serological surveys conducted in the southern region of Brazil had similar results, suggesting that age is a risk factor for detection of antibodies [76]. Comparable results are commonly seen in equine serosurveys not only for flaviviruses, but also for alphaviruses [27,28,77]. The evidence of exposure to SLEV and WNV observed in individuals aged 6 months or less, reported herein, suggests recent circulation of these flaviviruses. However, the detection of passively transferred maternal antibodies instead of exposure cannot be completely ruled out [78].

The negative results obtained when using molecular methods can be attributed to the absence of a current infection or to the brief and low viremia usually observed in dead-end hosts [79]. In the present study, no SLEV- or WNV-RNA was detected in serum, cerebrospinal fluid or CNS samples collected from horses with neurological disorders in RJ between 2015 and 2021. To increase the chances of detection, direct diagnostic methods using the serum samples of terminal hosts and collection of samples during the short period of viremia, which may peak before the onset of neurological clinical signs, should be performed [32]. However, it should be mentioned that SLEV-RNA has been detected by RT-PCR in brain samples of a horse with neurological disorder, and in a recent study, genetic evidence of WNV was found in equine red blood cells by portable nanopore sequencing [26,39]. Therefore, the negative molecular results presented here do not fully rule out the possibility that the infection with SLEV or WNV was the underlying cause of the neurological disorders of equines in RJ.

Epizootiological surveillance for arboviruses maintained in bird–mosquito cycles should always comprise not only molecular detection, but also serological investigation in local birds. Species of birds that act as amplifying hosts for WNV have long-lasting high-level viremia and persistent viral infection, and therefore, are easier to identify by molecular methods [7,80]. In 2019, during the epidemiological and epizootiological investigation of WNV in the state of Ceará, evidence of WNV exposure was found in distinct species of free-ranging passerines after the occurrence of a WNV fatal horse case [32]. In the present study, only horses were tested. The investigation of migratory species could also be particularly interesting for the detection of infection caused by arboviruses that occur in other regions. In Brazil, important migratory bird concentration sites exist. The Atlantic route is the main one and runs along the entire length of the Brazilian coast. In RJ, migratory bird stopovers include Lagoa da Ribeira and Lagoa Feia in the Norte Fluminense region and Restinga de Maçambaba and Ilha de Cabo Frio in the Coast mesoregion. Bird species migrate to South America and remain in the region from September to May [53,81].

## 5. Conclusions

Several reports on the circulation of WNV in Brazil emerged in the last decade, which could indicate the establishment of enzootic transmission cycles in the country. The higher prevalence for SLEV and WNV found in horses from the North Fluminense, Northwest Fluminense and Litoral mesoregions from RJ may also be related to local climatic and ecological conditions that favor enzootic cycles of transmission. The findings presented herein demonstrate the need to intensify the epidemiological surveillance of these arboviruses in Brazil and highlight the importance of future epizootiological investigations including both vectors and hosts, with particular interest in regions where cases of neurological syndromes have occurred in horses. The results obtained in this study imply SLEV and WNV previously circulated in horses from RJ, and should serve as an alarm to human and veterinary health professionals.

## Figures and Tables

**Table 1:** Number of properties in RJ where healthy horses were sampled between August 2015 and March 2017.

Regions	Municipalities	Properties	Horses Sampled Per Municipality	Total Horses Sampled Per Region
Metropolitan	Duque de Caxias Cachoeiras de Macacú Teresópolis	1 3 2	17 54 43	114
Northwest Fluminense	Bom Jesus do Itabapoana Itaperuna	1 1	38 42	80
North Fluminense	Campo dos Goytacazes São Fidelis Macaé	1 2 1	6 24 46	76
Coast	Araruama Saquarema Casimiro de Abreu	1 1 1	22 30 24	76
Centre Fluminense	Cantagalo Paraíba do Sul Areal	1 1 1	20 16 13	49
South Fluminense	Barra do Piraí Resende	2 1	17 23	40
Total:	16	21	435	435

**Table 2:** Number of properties and horses with neurological disorder in RJ sampled between August 2015 and May 2021 and tested for WNV and SLEV.

					Type and Number of Collected Samples				
Municipalities	Properties	Cases	Fatal Cases	Contact Cases	CSF	CNS Tissues	Spinal Cord	Serum	Horses Sampled
Teresópolis	2	13	2	10	1	0	0	23	23
Saquarema	1	1	1	10	1	1	1	11	11
Friburgo	1	1	1	0	0	0	0	1	1
Duas Barras	1	7	3	0	1	3	3	7	7
Maricá	1	3	2	22	2	2	2	25	25
Rio de Janeiro	1	5	2	0	0	0	0	5	5
06	07	30	11	42	5	06	06	72	72

**Table 3:** Prevalence of neutralizing antibodies for WNV and SLEV in horses from RJ, sampled between August 2015 and May 2021.

	PRNT_90_ Final Result						
	N (%)	WNV (%)	*p*-value	SLEV (%)	*p*-value	Undifferentiated flavivirus (%)	*p*-value
**Mesoregions**			0.39		<0.001		0.42
Metropolitan	114 (26.2)	0		11 (9.6)		6 (5.3)	
Northwest Fluminense	80 (18.4)	2 (2.5)		22 (27.5)		8 (10.0)	
North Fluminense	76 (17.5)	1 (1.3)		23 (30.0)		9 (11.8)	
Coast	76 (17.5)	2 (2.6)		18 (23.7)		5 (6.6)	
Centre Fluminense	49 (11.3)	0		15 (30.6)		2 (4.1)	
South Fluminense	40 (9.2)	0		0		2 (5.0)	
Function			<0.001		0.21		0.66
Sport	218 (50.1)	0		37 (17.0)		14 (6.4)	
Recreation	40 (9.2)	0		8 (20.0)		2 (5.0)	
Reproduction	175 (40.2)	4 (2.3)		44 (25.1)		16 (9.1)	
Work	2 (0.5)	1 (50.0)		0		0	
Sex			0.99		0.80		0.49
Female	310 (71.3)	4 (1.3)		62 (20.0)		25 (8.1)	
Male	125 (28.7)	1 (0.8)		27 (21.6)		7 (5.6)	
Age group			0.36		0.36		0.27
Group 1	7 (1.6)	0		2 (28.6)		0	
Group 2	63 (14.5)	0		11 (17.5)		2 (3.2)	
Group 3	130 (29.9)	0		20 (15.4)		7 (5.4)	
Group 4	141 (32.4)	3 (2.1)		34 (24.1)		13 (9.2)	
Group 5	94 (21.6)	2 (2.1)		22 (23.4)		10 (10.6)	

## Data Availability

Not applicable.

## Supplementary Materials

The following supporting information can be downloaded at: https://www.mdpi.com/article/10.3390/v14112459/s1, Table S1: Horses that were seropositive for WNV and SLEV in RJ. Table S2: Horses that were seropositive for undifferentiated flavivirus in RJ.

## References

[1] Pfeffer M., Dobler G. (2010). Emergence of zoonotic arboviruses by animal trade and migration. Parasit. Vectors.

[2] Casals J. (1957). Viruses: The versatile parasites; the arthropod-borne group of animal viruses. Trans. N. Y. Acad. Sci..

[3] Simmonds P., Becher P., Bukh J., Gould E.A., Meyers G., Monath T., Muerhoff S., Pletnev A., Rico-Hesse R., Smith D.B. (2017). ICTV Virus Taxonomy Profile: Flaviviridae. J. Gen. Virol..

[4] Travassos da Rosa A.P.A., Travassos da Rosa J.F.S., Pinheiro F.P., Vasconcelos P., Leão R.N.Q. (1997). Arboviroses. Doenças Infecc. e Parasitárias—Enfoque Amaz..

[5] Lima-Camara T.N. (2016). Emerging arboviruses and public health challenges in Brazil. Rev. Saude Publica.

[6] Diaz A., Coffey L.L., Burkett-Cadena N., Day J.F. (2018). Reemergence of St. Louis Encephalitis Virus in the Americas. Emerg. Infect. Dis..

[7] Komar N. (2003). West Nile virus: Epidemiology and ecology in North America. Adv. Virus Res..

[8] Castro-Jorge L.A., Siconelli M.J.L., Ribeiro B.D.S., Moraes F.M., Moraes J.B., Agostinho M.R., Klein T.M., Floriano V.G., Fonseca B.A.L.D. (2019). West Nile virus infections are here! Are we prepared to face another flavivirus epidemic?. Rev. Soc. Bras. Med. Trop..

[9] Weaver S.C., Barrett A.D. (2004). Transmission cycles, host range, evolution, and emergence of arboviral disease. Nat. Rev. Microbiol..

[10] Petersen L.R., Hayes E.B. (2008). West Nile virus in the Americas. Med. Clin. N. Am..

[11] Weaver S.C., Reisen W.K. (2010). Present and future arboviral threats. Antivir. Res..

[12] Liang G., Gao X., Gould E.A. (2015). Factors responsible for the emergence of arboviruses; strategies, challenges, and limitations for their control. Emerg. Microbes Infect..

[13] Saegerman C., Alba-Casals A., García-Bocanegra I., Dal Pozzo F., van Galen G. (2016). Clinical Sentinel Surveillance of Equine West Nile Fever, Spain. Transbound. Emerg. Dis..

[14] Kopp A., Gillespie T.R., Hobelsberger D., Estrada A., Harper J.M., Miller R.A., Eckerle I., Müller M.A., Podsiadlowski L., Leendertz F.H. (2013). Provenance and geographic spread of St. Louis encephalitis virus. mBio.

[15] Morales M.A., Barrandeguy M., Fabbri C., Garcia J.B., Vissani A., Trono K., Gutierrez G., Pigretti S., Menchaca H., Garrido N. (2006). West Nile virus isolation from equines in Argentina. Emerg. Infect. Dis..

[16] Chancey C., Grinev A., Volkova E., Rios M. (2015). The global ecology and epidemiology of West Nile virus. Biomed. Res. Int..

[17] Webster L.T., Fite G.L. (1933). A virus encountered in the study of material from cases of encephalitis in the St. Louis and Kansas City epidemics of 1933. Science.

[18] Lumsden L.L. (1958). St. Louis encephalitis in 1933; observations on epidemiological features. Public Health Rep..

[19] Beltrán F.J., Bechara Y.I., Guido G.G., Cicuttin G.L., Beaudoin J.B., Gury Dohmen F.E. (2014). Molecular detection of Saint Louis encephalitis virus in mosquitoes in Buenos Aires. Medicina (B Aires).

[20] Burton A.N., McLintock J., Francy D.B. (1973). Isolation of St. Louis Encephalitis and Cache Valley Viruses from Saskatchewan Mosquitoes. Can. J. Public Health = Revue Canadienne de Sante Publique.

[21] Causey O.R., Shope R.E., Theiler M. (1964). Isolation of St. Louis encephalitis virus from arthropods in Pará, Brazil. Am. J. Trop. Med. Hyg..

[22] de Souza Lopes O., de Abreu Sacchetta L., Coimbra T.L., Pereira L.E. (1979). Isolation of St. Louis encephalitis virus in South Brazil. Am. J. Trop. Med. Hyg..

[23] Pinheiro F.P., LeDuc J.W., Travassos da Rosa A.P., Leite O.F. (1981). Isolation of St. Louis encephalitis virus from a patient in Belém, Brazil. Am. J. Trop. Med. Hyg..

[24] Rocco I.M., Santos C.L., Bisordi I., Petrella S.M., Pereira L.E., Souza R.P., Coimbra T.L., Bessa T.A., Oshiro F.M., Lima L.B. (2005). St. Louis encephalitis virus: First isolation from a human in São Paulo State, Brazil. Rev. Inst. Med. Trop. Sao Paulo.

[25] Pinheiro F.P., Schatzmayr H., Travassos da Rosa A.P.A., Homma A., Bensabath G. (1975). Arbovirus antibodies in children of rural Guanabara, Brazil. Intervirology.

[26] Rosa R., Costa E.A., Marques R.E., Oliveira T.S., Furtini R., Bomfim M.R., Teixeira M.M., Paixão T.A., Santos R.L. (2013). Isolation of saint louis encephalitis virus from a horse with neurological disease in Brazil. PLoS Negl. Trop. Dis..

[27] Pauvolid-Corrêa A., Tavares F.N., Costa E.V., Burlandy F.M., Murta M., Pellegrin A.O., Nogueira M.F., Silva E.E. (2010). Serologic evidence of the recent circulation of Saint Louis encephalitis virus and high prevalence of equine encephalitis viruses in horses in the Nhecolândia sub-region in South Pantanal, Central-West Brazil. Mem. Inst. Oswaldo Cruz.

[28] Pauvolid-Corrêa A., Campos Z., Juliano R., Velez J., Nogueira R.M.R., Komar N. (2014). Serological Evidence of Widespread Circulation of West Nile Virus and Other Flaviviruses in Equines of the Pantanal, Brazil. PLoS Negl. Trop. Dis..

[29] Rodrigues S.G., Oliva O.P., Araújo F.A.A., Martins L.C., Chiang J.O., Henriques D.F., da Silva E.V.P., Rodrigues D.S.G., Prazeres A.S.C., Tavares-Neto J. (2010). Epidemiology of Saint Louis encephalitis virus in the Brazilian Amazon region and in the State of Mato Grosso do Sul, Brazil: Elevated prevalence of antibodies in horses. Rev. Pan-Amaz. Saúde.

[30] Silva J.R., Romeiro M.F., Souza W.M., Munhoz T.D., Borges G.P., Soares O.A., Campos C.H., Machado R.Z., Silva M.L., Faria J.L. (2014). A Saint Louis encephalitis and Rocio virus serosurvey in Brazilian horses. Rev. Soc. Bras. Med. Trop..

[31] Barbosa Costa G., Marinho P.E.S., Vilela A.P.P., Saraiva-Silva A.T., Crispim A.P.C., Borges I.A., Dutra A.G.S., Lobato Z.I.P., Dos Reis J.K.P., de Oliveira D.B. (2019). Silent Circulation of the Saint Louis Encephalitis Virus among Humans and Equids, Southeast Brazil. Viruses.

[32] Löwen Levy Chalhoub F., Maia de Queiroz-Júnior E., Holanda Duarte B., Eielson Pinheiro de Sá M., Cerqueira Lima P., Carneiro de Oliveira A., Medeiros Neves Casseb L., Leal das Chagas L., Antônio de Oliveira Monteiro H., Sebastião Alberto Santos Neves M. (2021). West Nile Virus in the State of Ceará, Northeast Brazil. Microorganisms.

[33] Weber M.N., Mosena A., Baumbach L.F., da Silva M.S., Canova R., Dos Santos D., Budaszewski R., de Oliveira L.V., Soane M.M., Saraiva N.B. (2021). Serologic evidence of West Nile virus and Saint Louis encephalitis virus in horses from Southern Brazil. Braz. J. Microbiol..

[34] Pauvolid-Corrêa A., Morales M.A., Levis S., Figueiredo L.T., Couto-Lima D., Campos Z., Nogueira M.F., da Silva E.E., Nogueira R.M., Schatzmayr H.G. (2011). Neutralising antibodies for West Nile virus in horses from Brazilian Pantanal. Mem. Inst. Oswaldo Cruz.

[35] Vieira M.A., Romano A.P., Borba A.S., Silva E.V., Chiang J.O., Eulálio K.D., Azevedo R.S., Rodrigues S.G., Almeida-Neto W.S., Vasconcelos P.F. (2015). West Nile Virus Encephalitis: The First Human Case Recorded in Brazil. Am. J. Trop. Med. Hyg..

[36] Silva A.S.G., Matos A.C.D., da Cunha M.A.C.R., Rehfeld I.S., Galinari G.C.F., Marcelino S.A.C., Saraiva L.H.G., Martins N.R.D.S., Maranhão R.P.A., Lobato Z.I.P. (2019). West Nile virus associated with equid encephalitis in Brazil, 2018. Transbound. Emerg. Dis..

[37] Martins L.C., Silva E., Casseb L., Silva S., Cruz A., Pantoja J., Medeiros D., Martins Filho A.J., Cruz E., Araújo M. (2019). First isolation of West Nile virus in Brazil. Mem. Inst. Oswaldo Cruz.

[38] Nota Técnica CEDESA n◦ 01/2019, 30 de agosto de 2019, Ocorrencia de Febre do Nilo Ocidental em Equino do Estado de Sao Paulo. Coordenadoria de Defesa Agropecuária, Secretaria de Agricultura e Abastecimento do Estado de São Paulo, Governo do Estado de São Paulo. https://www.defesa.agricultura.sp.gov.br/arquivos/sanidade-animal/nota-tecnica-febre-do-nilo-ocidental.pdf.

[39] Costa É.A., Giovanetti M., Silva Catenacci L., Fonseca V., Aburjaile F.F., Chalhoub F.L.L., Xavier J., Campos de Melo Iani F., da Cunha e Silva Vieira M.A., Freitas Henriques D. (2021). West Nile Virus in Brazil. Pathogens.

[40] Siconelli M.J.L., Jorge D.M.M., Castro-Jorge L.A., Fonseca-Júnior A.A., Nascimento M.L., Floriano V.G., Souza F.R., Queiroz- Júnior E.M., Camargos M.F., Costa E.D.L. (2021). Evidence for current circulation of an ancient West Nile virus strain (NY99) in Brazil. Rev. Soc. Bras. Med. Trop..

[41] Brasil (2004). Ministério da Saúde. Secretaria de Vigilância em Saúde. Segundo inquérito sorológico em aves migratórias e residentes do Parque Nacional da Lagoa do Peixe/RS para detecção do vírus da febre do Nilo ocidental e outros vírus. Bol. Eletrônico Epidemiológico.

[42] Melandri V., Guimarães A.É., Komar N., Nogueira M.L., Mondini A., Fernandez-Sesma A., Alencar J., Bosch I. (2012). Serological detection of West Nile virus in horses and chicken from Pantanal, Brazil. Mem. Inst. Oswaldo Cruz.

[43] Ometto T., Durigon E.L., de Araujo J., Aprelon R., de Aguiar D.M., Cavalcante G.T., Melo R.M., Levi J.E., de Azevedo Júnior S.M., Petry M.V. (2013). West Nile virus surveillance, Brazil, 2008–2010. Trans. R. Soc. Trop. Med. Hyg..

[44] Figueiredo L.T. (2000). The Brazilian flaviviruses. Microbes Infect..

[45] Figueiredo L.T. (2007). Emergent arboviruses in Brazil. Rev. Soc. Bras. Med. Trop..

[46] Guedes M.L.P. (2012). Culicidae (Diptera) No Brasil: Relações Entre Diversidade, Distribuição E Enfermidades. Oecologia Aust..

[47] Laporta G.Z., Ribeiro M.C., Ramos D.G., Sallum M.A. (2012). Spatial distribution of arboviral mosquito vectors (Diptera, Culicidae) in Vale do Ribeira in the South-eastern Brazilian Atlantic Forest. Cad Saude Publica.

[48] Schatzmayr H., Nogueira R.M., Travassos da Rosa A.P.A. (1986). An outbreak of dengue virus at Rio de Janeiro--1986. Mem. Inst. Oswaldo Cruz.

[49] Valle D., Pimenta D.N., Aguiar R. (2016). Zika, dengue and chikungunya: Challenges and issues. Epidemiol. Serv. Saude.

[50] Périssé A.R.S., Souza-Santos R., Duarte R., Santos F., de Andrade C.R., Rodrigues N.C.P., Schramm J.M.A., da Silva E.D., Jacobson L.D.S.V., Lemos M.C.F. (2020). Zika, dengue and chikungunya population prevalence in Rio de Janeiro city, Brazil, and the importance of seroprevalence studies to estimate the real number of infected individuals. PLoS ONE.

[51] Giovanetti M., de Mendonça M.C.L., Fonseca V., Mares-Guia M.A., Fabri A., Xavier J., de Jesus J.G., Gräf T., Dos Santos Rodrigues C.D., Dos Santos C.C. (2020). Yellow Fever Virus Reemergence and Spread in Southeast Brazil, 2016–2019. J. Virol..

[52] CEPERJ Fundação Centro Estadual de Estatística, Pesquisa e Formação de Servidores Públicos do Rio de Janeiro. Divisão Regional Segundo as Mesorregiões, Microrregiões Geográficas e Municípios. Estado do Rio de Janeiro—Posição e Extensão 2016. http://www.fesp.rj.gov.br/ceep/info_territorios/posicao_extencao.html.

[53] ICMCBio/Instituto Chico Mendes (2016). Relatório Anual de Rotas e Áreas de Concentração de aves migratórias no Brasil.

[54] Rappole J.H., Derrickson S.R., Hubálek Z. (2000). Migratory birds and spread of West Nile virus in the Western Hemisphere. Emerg. Infect. Dis..

[55] Chalhoub F.L.L. (2017). Investigação da Circulação dos Vírus da Encefalite de Saint Louis e do Oeste do Nilo em Equinos do Estado do Rio de Janeiro. Master’s Thesis.

[56] OpenEpi—Toolkit Shell for Developing New Applications 2019. https://www.openepi.com/SampleSize/SSPropor.htm.

[57] IBGE Censo Agropecuário Efetivo dos Rebanhos, por Tipo de Rebanho. @Ibgecomunica 2017. https://censoagro2017.ibge.gov.br//.

[58] Komar N. (2001). West Nile virus surveillance using sentinel birds. In: West Nile Virus: Detection, Surveillance, and Control. Ann. N. Y. Acad. Sci..

[59] Lanciotti R.S., Kerst A.J., Nasci R.S., Godsey M.S., Mitchell C.J., Savage H.M., Komar N., Panella N.A., Allen B.C., Volpe K.E. (2000). Rapid detection of west Nile virus from human clinical specimens, field-collected mosquitoes, and avian samples by a TaqMan reverse transcriptase-PCR assay. J. Clin. Microbiol..

[60] Lanciotti R.S., Kerst A.J. (2001). Nucleic acid sequence-based amplification assays for rapid detection of West Nile and St. Louis encephalitis viruses. J. Clin. Microbiol..

[61] (2021). RStudio. https://www.rstudio.com/products/rstudio/older-versions/.

[62] Mondini A., Bronzoni R.V., Cardeal I.L., dos Santos T.M., Lázaro E., Nunes S.H., Silva G.C., Madrid M.C., Rahal P., Figueiredo L.T. (2007). Simultaneous infection by DENV-3 and SLEV in Brazil. J. Clin. Virol..

[63] Terzian A.C., Mondini A., Bronzoni R.V., Drumond B.P., Ferro B.P., Cabrera E.M., Figueiredo L.T., Chiaravalloti-Neto F., Nogueira M.L. (2011). Detection of Saint Louis encephalitis virus in dengue-suspected cases during a dengue 3 outbreak. Vector Borne Zoonotic Dis..

[64] Romano-Lieber N.S., Iversson L.B. (2000). Serological survey on arbovirus infection in residents of an ecological reserve. Rev Saude Publica.

[65] Pauvolid-Corrêa A., Kenney J.L., Couto-Lima D., Campos Z.M., Schatzmayr H.G., Nogueira R.M., Brault A.C., Komar N. (2013). Ilheus virus isolation in the Pantanal, west-central Brazil. PLoS Negl. Trop. Dis..

[66] Bayeux J.J.M., Silva A.S.G., de Queiroz G.A., da Silva Santos B.S.Á., Rocha M.N., Rehfeld I.S., de Souza Franklin L.F., Valle L.B., Guedes M.I.M.C., Teixeira R.B.C. (2019). Epidemiological surveillance of West Nile virus in the world and Brazil: Relevance of equine surveillance in the context of “one health”. Braz. J. Vet. Res. Anim. Sci..

[67] Roehrig J.T., Hombach J., Barrett A.D. (2008). Guidelines for Plaque-Reduction Neutralization Testing of Human Antibodies to Dengue Viruses. Viral Immunol..

[68] Silva J.R., Medeiros L.C., Reis V.P., Chávez J.L., Munhoz T.D., Borges G.P., Soares O.A., Campos C.H., Machado R.Z., Baldani C.D. (2013). Serologic survey of West Nile virus in horses from Central-West, Northeast and Southeast Brazil. Mem. Inst. Oswaldo Cruz.

[69] Calisher C.H., Karabatsos N., Dalrymple J.M., Shope R.E., Porterfield J.S., Westaway E.G., Brandt W.E. (1989). Relações antigênicas entre flavivírus conforme determinado por testes de neutralização cruzada com anti-soros policlonais. J. Gen. Virol..

[70] Inouye S., Matsuno S., Tsurukubo Y. (1984). “Original antigenic sin” phenomenon in experimental flavivirus infections of guinea pigs: Studies by enzyme-linked immunosorbent assay. Microbiol. Immunol..

[71] Moraes M.M., Kubiszeski J.R., Vieira C.J.D.S.P., Gusmao A.F., Pratis T.S., Colombo T.E., Thies S.F., do Carmo Araujo F., Zanelli C.F., Milhim B.H.G.D.A. (2022). Detection of Saint Louis encephalitis virus in two Brazilian states. J. Med. Virol..

[72] Fritsch H., Pereira F.M., Costa E.A., Fonseca V., Tosta S., Xavier J., Levy F., Oliveira C.D., Menezes G., Lima J. (2022). Retrospective Investigation in Horses with Encephalitis Reveals Unnoticed Circulation of West Nile Virus in Brazil. Viruses.

[73] Beck C., Leparc-Goffart I., Desoutter D., Debergé E., Bichet H., Lowenski S., Dumarest M., Gonzalez G., Migné C., Vanhomwegen J. (2019). Serological evidence of infection with dengue and Zika viruses in horses on French Pacific Islands. PLoS Negl. Trop. Dis..

[74] Vorou R. (2016). Zika virus, vectors, reservoirs, amplifying hosts, and their potential to spread worldwide: What we know and what we should investigate urgently. Int. J. Infect. Dis..

[75] Burla R.S., Neto R.S., Werneck L.G., Maciel C.P., Silva R.A., Pessanha H.M., Oliveira V.D.P.S. (2013). Analysis of socioeconomic and environmental constraints to implement forestry in the Northern and Northwestern areas of Rio de Janeiro State. BOAARL.

[76] Vianna R.S.T. (2010). Inquéritos Soroepidemiológicos em Equinos da Região Sul do Brasil para Detecção de Anticorpos Anti-Flavivirus de Interesse em Saúde Pública. Master’s Thesis.

[77] Pauvolid-Corrêa A., Juliano R.S., Campos Z., Velez J., Nogueira R.M., Komar N. (2015). Neutralising antibodies for Mayaro virus in Pantanal, Brazil. Memórias Inst. Oswaldo Cruz.

[78] Jeffcott L.B. (1974). Some practical aspects of the transfer of passive immunity to newborn foals. Equine Vet. J..

[79] Pauvolid-Corrêa A., Varella R.B. (2008). Aspectos epidemiológicos da febre do Nilo Ocidental. Rev. Bras. Epidemiol..

[80] Blitvich B.J., Bowen R.A., Marlenee N.L., Hall R.A., Bunning M.L., Beaty B.J. (2003). Epitope-blocking enzyme-linked immunosorbent assays for detection of West Nile virus antibodies in domestic mammals. J. Clin. Microbiol..

[81] Valente R.M.S.J., Straube F.C., Nascimento J.L.X., Segtowick F. (2011). Conservação de Aves Migratórias Neárticas no Brasil.

