# Supplementary material for: Serological Evidence of Exposure to Saint Louis Encephalitis and West Nile Viruses in Horses of Rio de Janeiro, Brazil
Source: Viruses. 2022 Nov 6;14(11):2459. doi: 10.3390/v14112459 (PMC9695862; doi:10.3390/v14112459)
Supplement: Supplementary file 1 [file viruses-14-02459-s001.zip › viruses-1935881-supplementary.pdf]

Table S1 Horses that were seropositive for WNV and SLEV in RJ.

							PRNT90 titer					
**Sample ID**	Collection date	Sex	Activities	Age group	Mesoregions	Municipalities	WNV	SLEV	ZIKV	DENV-1	ILHV	Interpretation
**2**	January, 2016	F	Reproduction	G 5	North Fluminense	Macaé	<10	20	<10	<10	<10	SLEV
**6**	January, 2016	F	Reproduction	G 5	North Fluminense	Macaé	<10	20	<10	<10	<10	SLEV
**9**	January, 2016	F	Reproduction	G 4	North Fluminense	Macaé	10	40	<10	<10	<10	SLEV
**16**	January, 2016	F	Reproduction	G 5	North Fluminense	Macaé	10	40	<10	<10	<10	SLEV
**22**	January, 2016	F	Reproduction	G 4	North Fluminense	Macaé	<10	20	<10	<10	<10	SLEV
**23**	January, 2016	F	Reproduction	G 5	North Fluminense	Macaé	<10	80	<10	<10	<10	SLEV
**24**	January, 2016	F	Reproduction	G 5	North Fluminense	Macaé	320	40	<10	<10	<10	WNV
**29**	January, 2016	F	Reproduction	G 5	North Fluminense	Macaé	<10	40	<10	<10	<10	SLEV
**31**	January, 2016	F	Reproduction	G 5	North Fluminense	Macaé	<10	40	<10	<10	<10	SLEV
**34**	January, 2016	F	Reproduction	G 5	North Fluminense	Macaé	10	40	<10	<10	<10	SLEV
**38**	January, 2016	F	Reproduction	G 4	North Fluminense	Macaé	<10	20	<10	<10	<10	SLEV
**43**	January, 2016	F	Reproduction	G 3	North Fluminense	Macaé	10	160	<10	<10	<10	SLEV
**44**	January, 2016	F	Reproduction	G 4	North Fluminense	Macaé	<10	20	<10	<10	<10	SLEV
**45**	January, 2016	F	Reproduction	G 4	North Fluminense	Macaé	<10	80	<10	<10	<10	SLEV
**48**	March, 2016	F	Reproduction	G 4	North Fluminense	São Fidélis	<10	40	<10	<10	<10	SLEV
**51**	March, 2016	F	Reproduction	G 4	North Fluminense	São Fidélis	<10	160	<10	<10	<10	SLEV
**52**	March, 2016	F	Sports	G 2	North Fluminense	São Fidélis	10	40	<10	<10	<10	SLEV
**53**	March, 2016	F	Reproduction	G 3	North Fluminense	São Fidélis	10	80	<10	<10	<10	SLEV
**56**	March, 2016	F	Sports	G 4	North Fluminense	São Fidélis	<10	40	NA	NA	NA	SLEV

**59**	March, 2016	F	Sports	G 4	North Fluminense	São Fidélis	<10	20	<10	<10	<10	SLEV
**70**	March, 2016	M	Sports	G 2	North Fluminense	São Fidélis	<10	40	<10	<10	<10	SLEV
**71**	April, 2016	F	Reproducti on	G 4	North Fluminense	Campos dos Goytacazes	<10	160	<10	<10	<10	SLEV
**74**	April, 2016	M	Reproducti on	G 4	North Fluminense	Campos dos Goytacazes	20	80	<10	<10	<10	SLEV
**75**	April, 2016	M	Reproducti on	G 4	North Fluminense	Campos dos Goytacazes	10	160	<10	<10	<10	SLEV
**78**	April, 2016	F	Reproducti on	G 5	Northwest Fluminense	Bom Jesus do Itabapoana	10	80	<10	<10	<10	SLEV
**82**	April, 2016	F	Recreation	G 5	Northwest Fluminense	Bom Jesus do Itabapoana	<10	160	<10	<10	<10	SLEV
**87**	April, 2016	F	Recreation	G 1	Northwest Fluminense	Bom Jesus do Itabapoana	<10	40	<10	<10	<10	SLEV
**88**	April, 2016	F	Recreation	G 5	Northwest Fluminense	Bom Jesus do Itabapoana	<10	40	<10	<10	<10	SLEV
**90**	April, 2016	F	Recreation	G 4	Northwest Fluminense	Bom Jesus do Itabapoana	<10	80	<10	<10	<10	SLEV
**92**	April, 2016	f	Recreation	G 5	Northwest Fluminense	Bom Jesus do Itabapoana	<10	160	<10	<10	<10	SLEV
**96**	April, 2016	F	Sports	G 3	Northwest Fluminense	Bom Jesus do Itabapoana	10	40	<10	<10	<10	SLEV
**106**	April, 2016	F	Reproducti on	G 4	Northwest Fluminense	Bom Jesus do Itabapoana	<10	80	<10	<10	<10	SLEV
**108**	May, 2016	M	Sports	G 4	Northwest Fluminense	Itaperuna	<10	160	<10	<10	<10	SLEV
**109**	May, 2016	F	Reproducti on	G 5	Northwest Fluminense	Itaperuna	<10	80	<10	<10	<10	SLEV
**116**	May, 2016	F	Sports	G 4	Northwest Fluminense	Itaperuna	10	80	<10	<10	<10	SLEV
**121**	May, 2016	M	Sports	G 3	Northwest Fluminense	Itaperuna	10	160	<10	<10	<10	SLEV

**123**	May, 2016	M	Sports	G 2	Northwest Fluminense	Itaperuna	<10	320	<10	<10	<10	SLEV
**124**	May, 2016	F	Reproducti on	G 4	Northwest Fluminense	Itaperuna	<10	40	<10	<10	<10	SLEV
**125**	May, 2016	M	Sports	G 3	Northwest Fluminense	Itaperuna	<10	20	<10	<10	<10	SLEV
**126**	May, 2016	F	Sports	G 4	Northwest Fluminense	Itaperuna	<10	80	<10	<10	<10	SLEV
**127**	May, 2016	F	Sports	G 2	Northwest Fluminense	Itaperuna	<10	20	<10	<10	<10	SLEV
**129**	May, 2016	F	Sports	G 4	Northwest Fluminense	Itaperuna	<10	80	<10	<10	<10	SLEV
**131**	May, 2016	F	Sports	G 4	Northwest Fluminense	Itaperuna	<10	20	<10	<10	<10	SLEV
**133**	May, 2016	F	Reproducti on	G 5	Northwest Fluminense	Itaperuna	<10	40	<10	<10	<10	SLEV
**134**	May, 2016	F	Sports	G 2	Northwest Fluminense	Itaperuna	<10	20	<10	<10	<10	SLEV
**136**	May, 2016	F	Sports	G 2	Northwest Fluminense	Itaperuna	<10	20	<10	<10	<10	SLEV
**155**	March, 2017	F	Reproducti on	G 4	Northwest Fluminense	Bom Jesus do Itabapoana	256 0	10	<10	<10	<10	WNV
**156**	March, 2017	M	Work	G 5	Northwest Fluminense	Bom Jesus do Itabapoana	320	<10	<10	<10	<10	WNV
**163**	January, 2016	F	Sports	G 2	Coast	Araruama	<10	80	<10	<10	<10	SLEV
**172**	January, 2016	F	Reproducti on	G 4	Coast	Araruama	<10	160	<10	<10	<10	SLEV
**173**	January, 2016	F	Reproducti on	G 4	Coast	Araruama	40	<10	<10	<10	<10	WNV
**179**	January, 2016	F	Reproducti on	G 3	Coast	Casimiro de Abreu	<10	80	<10	<10	<10	SLEV
**182**	January, 2016	F	Reproducti on	G 3	Coast	Casimiro de Abreu	<10	20	<10	<10	<10	SLEV
**183**	January, 2016	F	Reproducti on	G 3	Coast	Casimiro de Abreu	<10	20	<10	<10	<10	SLEV
**186**	January, 2016	F	Reproducti on	G 4	Coast	Casimiro de Abreu	<10	40	<10	<10	<10	SLEV
**187**	January, 2016	F	Reproducti on	G 4	Coast	Casimiro de Abreu	<10	40	<10	<10	<10	SLEV

**189**	January, 2016	F	Reproduction	G4	Coast	Casimiro de Abreu	<10	40	<10	<10	<10	SLEV
**190**	January, 2016	F	Reproduction	G4	Coast	Casimiro de Abreu	10	80	<10	<10	<10	SLEV
**198**	January, 2016	F	Reproduction	G5	Coast	Casimiro de Abreu	10	80	<10	<10	<10	SLEV
**204**	January, 2016	M	Sports	G3	Coast	Saquarema	<10	80	<10	<10	<10	SLEV
**209**	January, 2016	F	Sports	G2	Coast	Saquarema	<10	20	<10	<10	<10	SLEV
**214**	January, 2016	F	Sports	G3	Coast	Saquarema	<10	40	<10	<10	<10	SLEV
**217**	January, 2016	F	Reproduction	G4	Coast	Saquarema	40	<10	<10	<10	<10	WNV
**220**	January, 2016	F	Reproduction	G3	Coast	Saquarema	10	40	<10	<10	<10	SLEV
**222**	January, 2016	F	Reproduction	G4	Coast	Saquarema	10	40	<10	<10	<10	SLEV
**223**	January, 2016	F	Reproduction	G4	Coast	Saquarema	10	80	<10	<10	<10	SLEV
**224**	January, 2016	F	Reproduction	G4	Coast	Saquarema	<10	160	<10	<10	<10	SLEV
**228**	January, 2016	F	Reproduction	G4	Coast	Saquarema	40	160	<10	<10	<10	SLEV
**238**	November, 2015	M	Reproduction	G5	Centre Fluminense	Cantagalo	<10	80	<10	<10	<10	SLEV
**240**	November, 2015	M	Reproduction	G 4	Centre Fluminense	Cantagalo	<10	20	<10	<10	<10	SLEV
**245**	November, 2015	M	Sports	G 3	Centre Fluminense	Cantagalo	<10	40	<10	<10	<10	SLEV
**247**	November, 2015	F	Sports	G 2	Centre Fluminense	Cantagalo	<10	40	<10	<10	<10	SLEV
**248**	November, 2015	M	Sports	G 3	Centre Fluminense	Cantagalo	80	320	<10	<10	<10	SLEV
**249**	November, 2015	M	Sports	G 1	Centre Fluminense	Cantagalo	<10	40	<10	<10	<10	SLEV
**255**	January, 2016	M	Sports	G 4	Centre Fluminense	Paraíba do Sul	20	80	<10	<10	<10	SLEV
**258**	January, 2016	M	Sports	G 4	Centre Fluminense	Paraíba do Sul	<10	20	<10	<10	<10	SLEV
**261**	January, 2016	M	Sports	G 4	Centre Fluminense	Paraíba do Sul	<10	320	<10	<10	<10	SLEV

**266**	January, 2016	F	Sports	G 4	Centre Fluminense	Paraíba do Sul	20	160	<10	<10	<10	SLEV
**269**	January, 2016	F	Reproduction	G 5	Centre Fluminense	Areal	10	40	<10	<10	<10	SLEV
**270**	January, 2016	F	Reproduction	G 5	Centre Fluminense	Areal	10	40	<10	<10	<10	SLEV
**275**	January, 2016	M	Recreation	G 5	Centre Fluminense	Areal	<10	160	<10	<10	<10	SLEV
**277**	January, 2016	F	Recreation	G 5	Centre Fluminense	Areal	<10	80	<10	<10	<10	SLEV
**278**	January, 2016	M	Recreation	G 5	Centre Fluminense	Areal	20	80	<10	<10	<10	SLEV
**285**	November, 2015	F	Sports	G 2	Metropolitan	Cachoeiras de Macacu	<10	20	<10	<10	<10	SLEV
**287**	November, 2015	M	Sports	G 5	Metropolitan	Cachoeiras de Macacu	<10	160	<10	<10	<10	SLEV
**311**	November, 2015	M	Sports	G 3	Metropolitan	Cachoeiras de Macacu	10	80	<10	<10	<10	SLEV
**322**	November, 2015	M	Sports	G 2	Metropolitan	Cachoeiras de Macacu	<10	20	<10	<10	<10	SLEV
**338**	May, 2016	M	Reproduction	G 3	Metropolitan	Duque de Caxias	<10	80	<10	<10	<10	SLEV
**345**	May, 2016	F	Reproduction	G 5	Metropolitan	Duque de Caxias	<10	20	<10	<10	<10	SLEV
**356**	August, 2015	M	Sports	G 3	Metropolitan	Teresópolis	<10	20	<10	<10	<10	SLEV
**358**	August, 2015	M	Sports	G 3	Metropolitan	Teresópolis	<10	20	<10	<10	<10	SLEV
**361**	August, 2015	M	Sports	G 3	Metropolitan	Teresópolis	10	80	<10	<10	<10	SLEV
**364**	August, 2015	M	Sports	G 3	Metropolitan	Teresópolis	<10	160	<10	<10	<10	SLEV
**372**	August, 2015	M	Sports	G 3	Metropolitan	Teresópolis	<10	20	<10	<10	<10	SLEV

WNV- West Nile virus; SLEV- Saint Louis encephalitis virus; ILHV- Ilheus virus; DENV-1- dengue virus serotype 1; ZIKV- Zika virus; F-Female; M-Male; G - Group

Table S2 Horses that were undifferentiated flavivirus in RJ.

							**PRNT90 titer**					
**Sample ID**	Collection date	Sex	Activities	Age group	Mesoregions	Municipalities	WNV	SLEV	ZIKV	DENV-1	ILHV	Interpretation

**1**	January, 2016	F	Reproduction	G 5	North Fluminense	Macaé	10	20	<10	<10	<10	Undifferentiated flavivirus
**17**	January, 2016	F	Reproduction	G 4	North Fluminense	Macaé	10	20	<10	<10	<10	Undifferentiated flavivirus
**25**	January, 2016	F	Reproduction	G 4	North Fluminense	Macaé	20	40	<10	<10	<10	Undifferentiated flavivirus
**26**	January, 2016	F	Reproduction	G 5	North Fluminense	Macaé	80	40	<10	<10	<10	Undifferentiated flavivirus
**32**	January, 2016	F	Reproduction	G 5	North Fluminense	Macaé	10	10	<10	<10	<10	Undifferentiated flavivirus
**33**	January, 2016	F	Reproduction	G 4	North Fluminense	Macaé	20	40	<10	<10	<10	Undifferentiated flavivirus
**60**	March, 2016	F	Sports	G 4	North Fluminense	São Fidélis	10	20	<10	<10	<10	Undifferentiated flavivirus
**64**	March, 2016	F	Reproduction	G 3	North Fluminense	São Fidélis	10	10	<10	<10	<10	Undifferentiated flavivirus
**72**	April, 2016	M	Reproduction	G 4	North Fluminense	Campos dos Goytacazes	20	20	<10	<10	<10	Undifferentiated flavivirus
**85**	April, 2016	F	Receration	G 4	Northwest Fluminense	Bom Jesus do Itabapoana	10	10	<10	<10	<10	Undifferentiated flavivirus
**151**	April, 2016	F	Reproduction	G 3	Northwest Fluminense	Bom Jesus do Itabapoana	10	10	<10	<10	<10	Undifferentiated flavivirus
**154**	April, 2016	F	Reproduction	G 4	Northwest Fluminense	Bom Jesus do Itabapoana	20	20	NA	NA	NA	Undifferentiated flavivirus
**111**	May, 2016	F	Sports	G 4	Northwest Fluminense	Itaperuna	20	40	<10	<10	<10	Undifferentiated flavivirus
**113**	May, 2016	F	Sports	G 5	Northwest Fluminense	Itaperuna	10	10	<10	<10	<10	Undifferentiated flavivirus
**119**	May, 2016	F	Sports	G 2	Northwest Fluminense	Itaperuna	20	40	<10	<10	<10	Undifferentiated flavivirus
**122**	May, 2016	M	Sports	G 4	Northwest Fluminense	Itaperuna	10	10	<10	<10	<10	Undifferentiated flavivirus
**128**	May, 2016	F	Sports	G 4	Northwest Fluminense	Itaperuna	10	10	<10	<10	<10	Undifferentiated flavivirus
**194**	January, 2016	F	Reproduction	G5	Coast	Casimiro de Abreu	10	10	<10	<10	<10	Undifferentiated flavivirus
**196**	January, 2016	F	Reproduction	G5	Coast	Casimiro de Abreu	10	10	<10	<10	<10	Undifferentiated flavivirus
**213**	January, 2016	F	Sports	G3	Coast	Saquarema	10	20	<10	<10	<10	Undifferentiated flavivirus
**215**	January, 2016	F	Sports	G3	Coast	Saquarema	20	10	<10	<10	<10	Undifferentiated flavivirus

**219**	January, 2016	F	Reproduction	G4	Coast	Saquarema	80	40	<10	<10	<10	Undifferentiated flavivirus
**262**	January, 2016	M	Sports	G 4	Centre Fluminense	Paraíba do Sul	10	10	<10	<10	<10	Undifferentiated flavivirus
**279**	January, 2016	F	Recreation	G 5	Centre Fluminense	Areal	10	10	<10	<10	<10	Undifferentiated flavivirus
**310**	November, 2015	F	Sports	G 3	Metropolitan	Cachoeiras de Macacu	40	40	<10	<10	<10	Undifferentiated flavivirus
**328**	November, 2015	M	Sports	G 2	Metropolitan	Cachoeiras de Macacu	20	40	<10	<10	<10	Undifferentiated flavivirus
**329**	November, 2015	F	Reproduction	G 4	Metropolitan	Cachoeiras de Macacu	20	40	<10	<10	<10	Undifferentiated flavivirus
**337**	May, 2016	F	Reproduction	G 5	Metropolitan	Duque de Caxias	10	10	<10	<10	<10	Undifferentiated flavivirus
**353**	August, 2015	M	Sports	G 3	Metropolitan	Teresópolis	10	10	<10	<10	<10	Undifferentiated flavivirus
**373**	August, 2015	M	Sports	G 3	Metropolitan	Teresópolis	20	20	<10	<10	<10	Undifferentiated flavivirus
**429**	May, 2016	M	Sports	G 5	South Fluminense	Resende	80	40	<10	<10	<10	Undifferentiated flavivirus
**435**	May, 2016	F	Reproduction	G 5	South Fluminense	Resende	40	40	<10	<10	<10	Undifferentiated flavivirus

WNV- West Nile virus; SLEV- Saint Louis encephalitis virus; ILHV- Ilheus virus; DENV-1- dengue virus serotype 1; ZIKV- Zika virus; F-Female; M-Male; G - Group
